# Alveolar soft part sarcoma of lung: report of a unique case with emphasis on diagnostic utility of molecular genetic analysis for TFE3 gene rearrangement and immunohistochemistry for TFE3 antigen expression

**DOI:** 10.1186/s13000-015-0399-5

**Published:** 2015-09-15

**Authors:** Ming Zhao, Qiu Rao, Cuiyun Wu, Zhongsheng Zhao, Xianglei He, Guoqing Ru

**Affiliations:** Depatment of Pathology, Zhejiang Provincial People’s Hospital, Hangzhou, 310014 China; Department of Pathology, Nanjing Jinling Hospital, Nanjing, 210000 China; Department of Radiology, Zhejiang Provincial People’s Hospital, Hangzhou, 310014 China

**Keywords:** Alveolar soft part sarcoma, ASPS, Lung, TFE3, FISH, Differential diagnosis

## Abstract

Alveolar soft part sarcoma (ASPS) is a rare, malignant mesenchymal tumor of distinctive clinical, morphologic, ultrastructural, and cytogenetical characteristics. It typically arises in the extremities of adolescents and young adults, but has also been documented in a number of unusual sites, thus causing diagnostic confusions both clinically and morphologically. The molecular signature of ASPS is a specific der(17)t(X;17)(p11.2;q25) translocation, which results in the fusion of TFE3 transcription factor gene at Xp11.2 with ASPL at 17q25. Recent studies have shown that the ASPL-TFE3 fusion transcript can be identified by reverse-transcriptase polymerase chain reaction analysis and TFE3 gene rearragement can be detected using a dual-color, break apart fluorescence in situ hybridization assay in paraffin-embedded tissue, and the resultant fusion protein can be detected immunohistochemically with antibody directed to the carboxy terminal portion of TFE3. Herein, we report a unique case of ASPS presenting as an asymptomatic mass in the lung of a 48 year-old woman without evidence of a primary soft tissue tumor elsewhere at the time of initial diagnosis. To the best of our knowledge, this is the third report of such cases appearing in the English language literature to date. We emphasize the differential diagnoses engendered by ASPS including a series of tumors involving the lung that have nested and alveolar growth patterns, and both clear and eosinophilic cytoplasm, and demonstrate the utility of molecular genetic analysis for TFE3 rearrangement and immunohistochemistry for TFE3 antigen expression for arriving at accurate diagnosis.

## Background

Alveolar soft part sarcoma (ASPS) is a rare mesenchymal neoplasm with a highly distinctive histologic appearance, ultrastructure, and cytogenetic profile involving a non-reciprocal t(X;17)(p11.2;q25) [1, 2]. The translocation fuses the TFE3 transcription factor gene at Xp11.2 to ASPL (ASPSCR1), a novel gene on chromosome 17q25 and presents as type 1 and 2 variants involving the fusion of the first seven exons of the ASPL gene to exon 6 (type 1) or 5 (type 2) of the TFE3 transcription factor gene [3]. ASPS most commonly occurs in the deep soft tissue of lower extremities in adolescents and young adults, or the head and neck region, especially the tongue and orbit in infants and children [1, 2], but has also been occasionally reported in a variety of unusual locations including the lung, stomach, liver, breast, larynx, heart, urinary bladder, and female gential tract [4–10]. When presented at these unusual sites ASPS may significantly causes diagnostic challenges due to its histologic overlap with a number of primary or secondary neoplasms ocurring in those sites. Herein, we present a case of ASPS occurring primarily in the lung in a 48 year-old woman without evidence of a primary soft tissue tumor elsewhere at the time of initial diagnosis.

## Case presentation

**Fig. 1:**
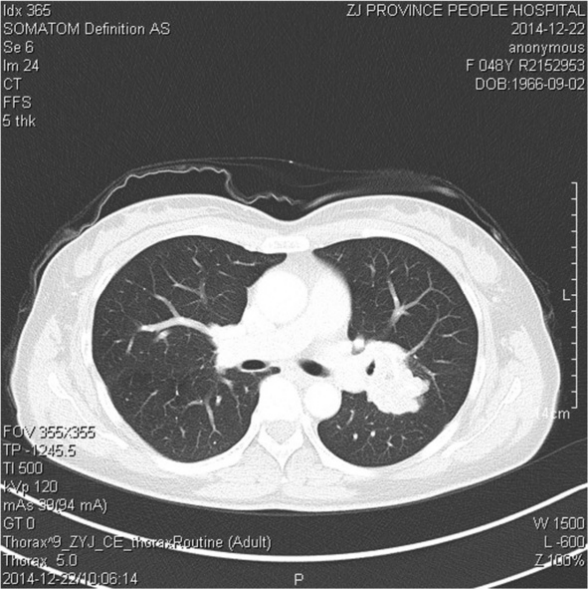
Radiology of ASPS of the lung. Computed tomograph (CT) scan demonstrated a well-demarcated, lobulated, heterogeneously enhanced mass located at the level of hilum of left lung

A previouly healthy 48 year-old female patient was incidentally identified to have a left-lung mass on thoracic radiology for routine medical examination. Subsequent computed tomograph (CT) scan demonstrated a well-demarcated, partly lobulated, heterogeneously enhanced mass measuring of 3.8 × 3.7 cm, located at the hilum of left lung (Fig. 1). A lung cancer was suggested. Precutaneous needle biopsy of the mass revealed no evidence of maligangce was noted. Left pneumonectomy with hilar and mediastinal lymphadenectomy was performed and no additional therapy was administered. Neither a history of a remote tumor nor other soft tissue or visceral lesions was discovered on the patient. A follow-up 12 months after the initial surgery found the patient to be at a good status with no evidence of tumor recurrence or metastasis.

The resection specimen was fixed in 10 % buffered formalin. Tissue sections were routinely processed and stained with hematoxylin and eosin. IHC analysis was performed using advidin-biotin-complex immunoperoxidase technique with a panel of commercially available primary antibodies to the following antigens: cytokeratin AE1/AE3 (AE1/3, Dako, Denmark), cytokeratin 7 (CK7) (OV-TL12/30, Dako), cytokeratin 20 (CK20), high molecular weight cytokeratin (34βE12, Dako), vimentin (V9, Dako), Thyroid transcription factor 1 (TTF1) (8G7G3/1, Dako), NapsinA (polyclonal, Dako), CD10 (56C6, Dako), PAX8 (polyclonal, Proteintech, China), smooth muscle actin (SMA) (1A4, Dako), desmin (D33, Dako), TFE3 (polyclonal, Abcam, UK), melan-A (A103, Dako), HMB45 (HMB45, Dako), S100 protein (polyclonal, Dako), chromogranin (polyclonal, Dako), synaptophysin (polyclonal, Dako), Hepatocyte paraffin-1 (HepPar-1) (OCH1E5, Dako), CD34 (QBEnd/10, Dako), myogenin (MyF4, Dako) and Ki67 (MIB-1, Dako). Appropriate positive and negative controls were run concurrently for all the markers tested. FISH assay for TFE3 gene rearrangement [11] and RT-PCR amplification and DNA sequence analysis for ASPL-TFE3 fusion transcript [12] were performed according to previously had been suggested. For ASPL-TFE3 fusion FISH assay, the bacterial artificial chromosome (BAC) clones RP11-765O14 (195 kb) and RP11-665 F9 (176 kb), located centromeric to the ASPL gene locus, were labeled with 5-fluorescein dUTP. The BAC clones RP11-416B14 (182 kb) and RP11-344 N17 (202 kb), located telomeric to the TFE3 gene locus, were labeled with 5-ROX-dUTP. RT-PCR analysis for ASPL-TFE3 fusion transcript was performed using Qiagen OneStep RT-PCR kit (Qiagen, German). Primer sequences uesd as followings: ASPL-E7-F3: TCCAAGCCAAAGAAGTCC; TFE3-E6-R1: TCAAGCAGATTCCCTGACAC. DNA sequencing of PCR products was performed using a Qiagen 3000 BioRobot (Qiagen, German). Written informed consent was obtained from the patient.

**Fig. 2:**
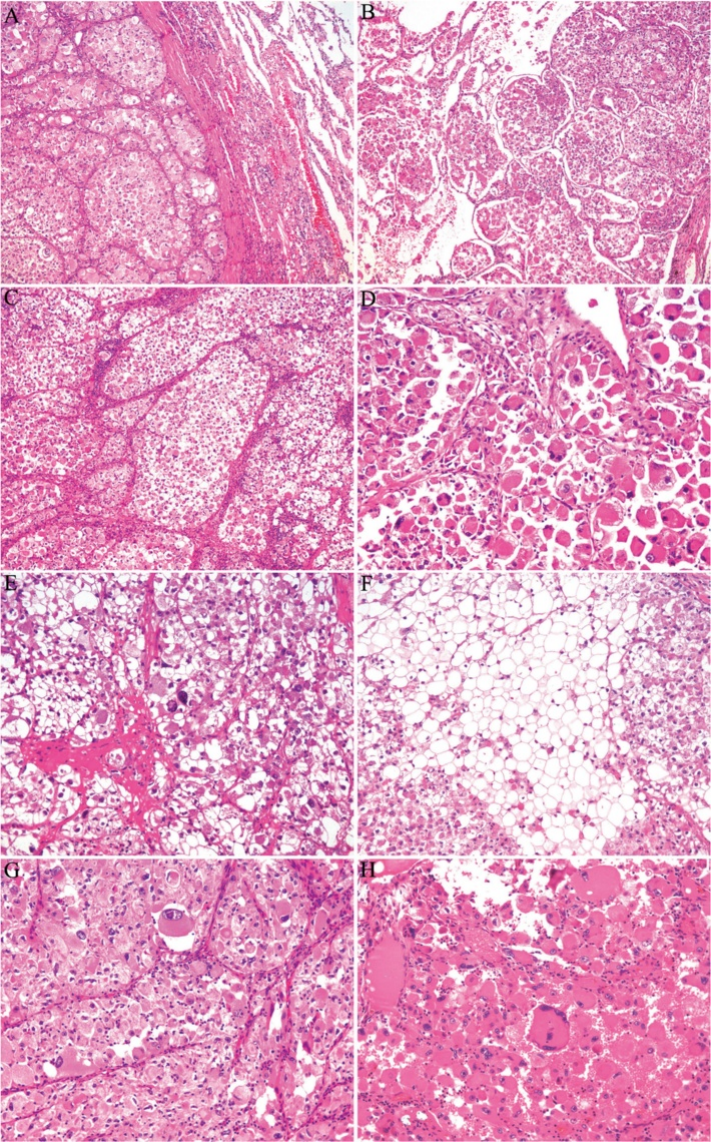
Microscopical features of ASPS of the lung. **a** and **b** The tumor overall demonstrated an expansile growth pattern with focally infiltrating into the alveolar spaces. **c** The tumor was composed of nests and alveolus and separated by thin-walled fibrous septa that contained abundant vascular networks and lymhoplasmacytic infiltrations. **d**-**f** The tumor cells were large, polygonal to round, and discohesive with voluminous eosinophilic, to pale, to clear cytoplasm. **g** Pleomorphic nuclei with prominent eosinophilic nucleoli. H: Multinucleation

**Fig. 3:**
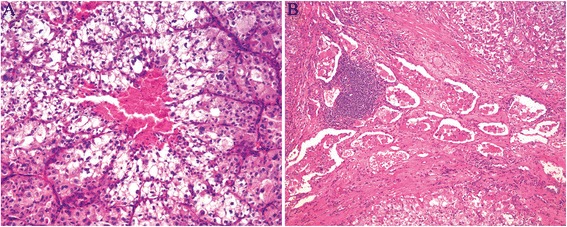
Microscopical features of ASPS of the lung. **a** and **b** Foci of microscopic necrosis and vascular tumor invasion were noted

Macroscopic examination of the resected specimen revealed a circumscribed but noncapsulated, lobular, firm tumor of white-to-gray color that measured 3.5 × 3.2 × 3.0 cm, and arised from the bronchus with lateral extension towards subpleural areas without gross evidence of pleural contracture. Microscopically, the tumor overall demonstrated an expansile and vaguely lobular growth pattern (Fig. 2a) with limited invasive fronts where tumor cells infiltrated focally into the alveolar spaces (Fig. 2b) and bronchic cartilage. The tumor was composed of predominantly of variable-sized nests and alveolus, separated by thin-walled fibrous septa that contained abundant vascular networks and prominent lymhoplasmacytic infiltrations (Fig. 2c). In tiny areas, a trabecular arrangement, reminiscent of hepatocellular carcinoma (HCC), was also noted. Cytologically, the tumor cells were large, polygonal to round, and often discohesive with voluminous deeply eosinophilic, to pale, finely granular eosinophilic, to clear cytoplasm and distinct borders (Fig. 2d-f). Frequently, the eosinophilic cytoplasm of the cells shrunk and condensed away from the membrane to the nucelus, creating an appearance resembling the so-called “spider cells” commonly seen in epithelioid perivascular epithelioid cell tumor (PEComa), or rhabdoid cells. The nuclei were eccentrically placed and were small, round with inconspicuous nucleoli, to markedly enlarged, pleomorphic with vesicular chromatin and prominent eosinophilic nucleoli (Fig. 2g). Multinucleation was occasionally obseverd (Fig. 2h), mitoses were scarce. Foci of microscopic necrosis and vascular tumor invasion were noted (Fig. 3a-b). Evidence of any squamous or cylindrical cell abnormalities or epithelial tumor was lacking. The hilar and mediastinal lymph nodes were free of tumor.

**Fig. 4:**
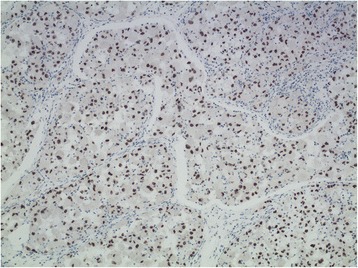
Immunohistochemical features of ASPS of the lung. By immunohistochemistry, the tumor cells showed diffuse and strong nucler positivity for TFE3

**Fig. 5:**
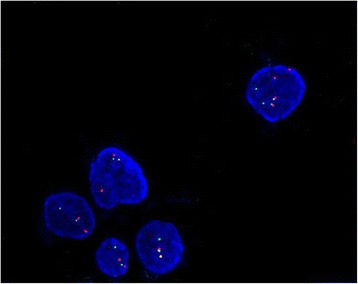
Molecular genetic features of ASPS of the lung. TFE3 fluorescence in situ hybridization assay showed 1 set of fusion signal and 1 set of green and red split signal, indicating evidence of a TFE3 gene rearrangement

**Fig. 6:**
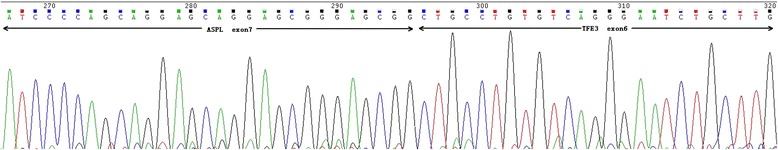
Molecular genetic features of ASPS of the lung. RT-PCR amplification and DNA sequence analysis identified a type 1 ASPL-TFE3 fusion transcript within the tumor

By IHC, the tumor cells showed diffuse and strong nucler positivity for TFE3 (Fig. 4) but negativity for all the other markers detected except for Ki67, which labeled approximate 10 % tumor cells. FISH assay of the tumor cells showed a single interphase nucleus with split red and green signals observed in approximate 15 % tumorous nuclei, indicating the presence of a TFE3 gene rearrangement involving X chromosome (Fig. 5). RT-PCR amplification and DNA sequence analysis identified a type 1 ASPL-TFE3 fusion transcript with fusion of the first seven exons of the ASPL gene to exon 6 of the TFE3 transcription factor gene (Fig. 6). A diagnosis of primary ASPS of the lung was rendered on the basis of exclusion of a secondary ASPS and common and not-so-common differential diagnoses of tumors reported in the lung, and on the basis of morphology, IHC (TFE3 positivity), and molecular genetics (TFE3 gene rearrangement) supportive of ASPS.

## Discussion

ASPS is a rare malignant mesenchymal tumor accounting for less than 1 % of all soft tissue tumors [2]. Clinically, it typically presents as a soft, painless, slow-growing mass and most classically occurs in the deep soft tissue of the extremities in adolescents and young adults (15–35 y of age), with a female predominance [1, 2]. The most common locations include buttocks/thighs, legs/popliteal fossa, chest wall/trunk, and the upper extremities. In children and infants, the head and neck region including the tongue and orbit, is a common location. Unusual primary soft tissue locations include the retroperitoneum, mediastinum, and bone [4, 5]. Visceral organ, such as lung, liver and brain, involving by ASPS mostly represents a metastasis from a primary soft tissue tumor elsewhere. Hovever, sporadic reports have certainly documented primary ASPS of visceral organs including the lung, stomach, liver, breast, larynx, heart, urinary bladder, and female gential tract [8, 7, 6, 10, 9, 13]. Primary ASPS of lung is extraordinarily rare, to the best knowledge of us, our case represents only the third one of such cases that have been reported in the English language literature since the mid 1960’s [14, 8]. The two previously reported cases were from Japan, and Korea, respectively. However, the Japanese case [14], which was initially described as a tumor arising from the pulmonary vein at the lung hilus, had been questioned by other authors as a tumor origining in the mediastinum rather than in the lung [8]. In contrast to metastatic ASPS of the lung that often radiologically appeared as mutiple and bilateral nodules, both our and the Corean case [8] presented as a solitary, asymptomatic mass in the lung, similar to primary ASPS that had been reported in other visceral organs.

Histologically, ASPS mostly presents stereotypical morphologic features with round nests and alveoli composed of dyscohesive uniform polygonal neoplastic cells having round nuclei with vesicular chromatin, a prominent nucleolus, and abundant cytoplasm containing PAS positive, distase resistant crystals [2, 1]. However, the organoid appearance may be lacking and the tumor may be composed of sheets of neoplastic cells. Rarely, ASPS may shows light microscopic features that depart from the conventional morphology and cause differential diagnostic confusions. Our case showed several unusual morphologic features of ASPS that have only been occasionally mentioned in the literature, including heavy lymphocytic infiltrate, anaplasia, clear cells, rhabdoid-like cells, and multinucleation [1, 13].

The cell of origin or, better, line of differentiation taken by ASPS is elusive, and attempts to investigate it by ultrastructural and immunohistochemical studies have failed to elucidate the line of differentiation, with controversial results [2, 1]. Recently, the molecular signature of ASPS has been described as a specific unbalanced translocation: der(17)t(X;17)(p11.2;q25) [15]. This translocation results in the fusion of TFE3 transcription factor gene at Xp11.2 with ASPL at 17q25 [3, 12]. Recent studies has shown that the ASPL-TFE3 fusion transcript can be identified by RT-PCR analysis and TFE3 gene rearragement can be detected using a dual-color, break apart FISH assay in paraffin-embedded tissue, both can be uesd as powerful tools for diagnosis of ASPS [12, 16, 17], in addition, the resultant fusion protein can be detected by IHC with an antibody directed to the carboxy terminal portion of TFE3 with high sensitivity and specificity [18]; all the three tools were used in the current case to confirm the diagnosis of ASPS in the lung. TFE3 gene rearrangment by FISH assay and moderate to strong nuclear TFE3 positivity by IHC are virtually pathognomonic for ASPS, Xp11.2 translocation associated RCC [19], and a subset of PEComa that harbors TFE3 gene fusion [20]. Xp11.2 translocation associated RCC is a recently described category of renal tumor that is characterized by a papillary architecture composed of cells with voluminous clear or eosinophilic cytoplasm and psammoma bodies. Genetically, Xp11.2 translocation associated RCC harbors a balanced t(X;17)(p11.2;q25) translocation in the majority cases, which is in contrast to that of ASPS [19, 21]. TFE3 gene fusion associated PEComa, a most recently described subtype of PEComa that occurs primarily in young adults of both renal and extrarenal, and features of prominent epithelioid cells with alveolar architecture, as well as an aggressive clinical course [20]. FISH assay and RT-PCR analysis in these tumors have shown TFE3 gene rearrangement and amplification, respectively. However, the partner fusion gene of TFE3 in PEComa is largely unknown nowadays [22, 23]. Distinguishing these tumors may need IHC for additional markers (as discussed below).

Although its high sensitivity and specificity for identification of neoplasms with associated gene fusion, detection of TFE3 reactivity by IHC has been shown to be technically difficult, not inrequently accompanied with strong background stain, or even with false positive and negative results [18]. In addition, significant TFE3 expression can ocassionally be seen in tumors that not harbor an associated gene fusion, such as granular cell tumor [24], paraganglioma [12], and adrenocortical carcinoma [12], these findings are of particular importance since all these tumors may show overlapping morphological features with ASPS.

The differential diagnosis in the current case is relatively broad that includes the rhabdoid or large cell undifferentiated lung carcinoma [25], paraganglioma [26], epithelioid PEComa (clear cell sugar tumor) [27], malignant granular cell tumor [28], melanoma, and metastatic carcinoma such as RCC, adrenocortical carcinoma, and HCC [29]. Although careful histomorphologic investigation obviously plays a critical role in this differential diagnosis, IHC, and occasionally molecular genetic analysis will prove decisive, as evidenced by the current case. Briefly, carcinomas of pulmonary origin would be expected to show considerable CK expression in most cases, whereas ASPS does not express CK. Metastatic Xp11.2 translocation associated RCC may show only weak CK expression, but generally show strong PAX8 nuclear expression, a finding not seen in ASPS. Metastatic adrenocortical carcinoma and HCC would be expected to show MelanA and HepPar-1 expression in the majority of cases, respectively, whereas ASPS expresses neither of the two markers. Paraganglioma, but not ASPS, expresses neuroendocrine markers, such as chromogranin A and synaptophysin. Granular cell tumor and melanoma typically display strong, uniform S100 protein expression, which is absent in ASPS. Expression of melanocytic markers, such as HMB45 and MelanA, would be seen in melanoma and epithelial PEComa, but not in ASPS.

## Conclusions

In summary, we report a unique case of primary ASPS of the lung. Because of its unusual anatomic presentation, problems in diagnosis may arise. A detailed clinical history, histomorphology, as well as immunohistochemical and molecular genetic studies may help separate this tumor from other more common primary pulmonary and metastatic neoplasms. This case highlights the ubiquitous distribution of this tumor and the need to consider this neoplasm in the differential diagnosis of primary pulmonary lesions.

## Consent

Written informed consent was obtained from the patient for publication of this case report and accompanying images. A copy of the written consent is available for review by the Editor-in Chief of this Journal.
